# Easy to Read Health Education Material Improves Oral Health Literacy of Older Adults in Rural Community-Based Care Centers: A Quasi-Experimental Study

**DOI:** 10.3390/healthcare9111465

**Published:** 2021-10-29

**Authors:** Kuo-Ting Sun, Tzong-Ming Shieh, Shih-Min Hsia, Valendriyani Ningrum, Xin-Yi Lin, Yin-Hwa Shih

**Affiliations:** 1 Department of Pediatric Dentistry, China Medical University Hospital, Taichung 40402, Taiwan; ktsun@mail.cmu.edu.tw; 2 School of Dentistry, China Medical University, Taichung 40402, Taiwan; tmshieh@mail.cmu.edu.tw; 3 Graduate Institute of Metabolism and Obesity Sciences, Taipei Medical University, Taipei 110301, Taiwan; bryanhsia@tmu.edu.tw; 4 School of Nutrition and Health Sciences, Taipei Medical University, Taipei 110301, Taiwan; 5 School of Dentistry, Baiturrahmah University, By Pass Km 15 Aie Pacah, Padang 25586, Indonesia; valend888@gmail.com; 6 Department of Healthcare Administration, Asia University, Taichung 41354, Taiwan; shinilinsheng@yahoo.com.tw

**Keywords:** easy to read, health education material, older adults, community-based care center, oral health literacy

## Abstract

Health education increases older adults’ health knowledge and affects their health outcomes. Older adults have physical changes with aging, such as blurred vision and cognitive decline. Therefore, health education materials must be legible in their case. This study, following the “easy (EZ) to read” concept, designed oral health education material and tested the learning effectiveness of older adults in rural community-based care centers in central Taiwan. Three of the communities were provided EZ to read health education material (*n* = 72), while three were given general text material (*n* = 57) as the control group. We collected pre-test and post-test scores using the Mandarin version of the oral health literacy adult questionnaire (MOHL-AQ). The demographic background of the EZ to read and general text groups showed no significant difference (*p* > 0.05). Analysis of covariance (ANCOVA) showed that the EZ to read material significantly improved total scores of oral health literacy (*p* < 0.001). The chi-square test showed a significant improvement in oral health literacy levels (*p* < 0.001). We suggest applying EZ to read concepts to widen the field of older adult education and to reduce illegibility-induced health knowledge disparities.

## 1. Introduction

Rapid urbanization and aging are two inevitable and intersecting demographic trends of the 21st century. By 2030, it is estimated that more than one in eight people around the world will be 65 years or older [1]. According to the World Health Organization’s (WHO) strategy for global aging [2], urban areas should build age-friendly infrastructure and provide health programs by leveraging innovative medical technology. However, rural communities are home to a higher proportion of older adults, and overlooking rural healthcare services and infrastructure creates a situation of health information inequity for these older residents [3,4].

In rural areas, older adults receive health information from social media, medical professionals in the hospital, and health promotion activities in the community [5]. Physical changes due to aging, such as blurred vision and cognitive decline as well as the low educational level of rural older adults, make health education materials illegible for many of them, impeding them from receiving health information [6]. Therefore, the design of educational materials should consider their specific requirements. Such individuals need enhanced materials with larger sized words, more images, and shorter sentences [7].

“Easy (EZ) to read” is a method of presenting written information so that it is easier to understand for people with difficulty reading. It advocates short sentences, expresses ideas in a small number of simple words, prefers sentences in the active voice, suggests that words be present in at least 14-point text, and recommends that documents use a sans-serif font [8]. Health literacy is the degree to which individuals have the capacity to obtain, process, and understand basic health information needed to make appropriate health decisions. Applying EZ to read concepts into health education materials significantly increases readers’ knowledge [9] and health literacy [10].

Older adults also have different dental problems from those of the younger population. They have reduced saliva production and frequent tooth loss, and most of them have full or partial dentures [11]. Apart from the often-reported sex, educational level, and financial status, health literacy and the quality of patient–dentist communication are also essential factors in improving oral health in the population [12,13].

In Taiwan, community-based care centers take care of adults aged 65 years and over with normal ADL (activity of daily living) functions. The centers provide health promotion and disability prevention activities, periodic health education courses, and meals that meet the nutritional needs of older adults. Community-based care centers play an important role in health promotion in rural communities. Therefore, we would like to investigate the learning effectiveness of EZ to read health education materials on improvement of oral health literacy among older adults in rural community-based care centers.

## 2. Materials and Methods

### 2.1. Study Design and Setting

This was a quasi-experimental study. We used convenience sampling to collect data at six community-based care centers in the rural areas of central Taiwan near Asia University (Taichung City, Wufeng District and Caotun Town, Nantou County). We included older adults aged 60 and above who had literacy skills (reading and writing ability) and spoke Mandarin Chinese and Taiwanese Hokkien. Of these six centers, we randomly assigned three to use the EZ to read oral health education material (72 participants in Jia Lao, Tonglin, and Dunhe) and assigned general text oral health education material for the other three (57 participants in Shanglin, Wufeng, and Bifeng). The researcher attended the community-based care center at 9 o’clock in the morning. The material was used in a health education course of approximately 30 min, accompanied by PowerPoint slides. The content of the PowerPoint slides was the same as the content in the educational material. All the participants were asked to complete a pre-test before the course and a post-test after the course using the Mandarin version of the oral health literacy adult questionnaire (MOHL-AQ). The return rate of the questionnaire was 100%.

### 2.2. Social Demographic Data

Demographic information comprising age, sex, educational level, living status, denture-wearing status and type, and regular dental visits for denture adjustment was collected. Individuals with missing data were excluded from statistical analysis.

### 2.3. The Mandarin Version of the Oral Health Literacy Adult Questionnaire (MOHL-AQ)

The MOHL-AQ was published by Ho et al. (2019) [14]. The overall content validity is 95%; inter-rater reliability is 98%, and Cronbach’s α coefficient value is 0.77. This questionnaire has 17 items across 4 domains: reading comprehension, numeracy, listening (communication skills), and decision-making. A correct answer to each item earns one point. Scores of 0–9 points indicate an inadequate level; 10–11 points indicate a marginal level; and 12–17 points indicate an adequate level of oral health literacy.

### 2.4. Educational Material

We designed the content of the educational material according to the questions of the MOHL-AQ. The EZ to read material was designed following the guidelines in the article published by Daghio et al. (2006) [15]. We used 26-point (5.65 mm high) DFKai-SB font, with a spacing of 1.5, and added a picture to the right side of the text. The general text material used 14-point DFKai-SB font, single spacing, and no pictures.

### 2.5. Statistical Analysis

Statistical analyses were conducted using SPSS 25 (IBM, Armonk, NY, USA). The demographic background and the pre-test and post-test MOHL-AQ levels between the two groups were analyzed using the chi-square test. The pre-test and post-test scores of the two groups were analyzed using ANCOVA. Statistical significance was set at α = 0.05.

## 3. Results

### 3.1. Similar Demographic Background between EZ to Read Group and General Text Group

The demographic data showed no significant differences in sex, age, educational level, living status, denture status, denture type, or regular dental visit for denture adjustment between the EZ to read group and the general text group (Table 1). The demographic context of these two groups was well controlled to increase the reliability of the ANCOVA analysis.

### 3.2. The ANCOVA Analysis Showed Significant Score Improvements in EZ to Read Group

The post-test score of the EZ to read group was 11.22 (3.55), while that of the general text material group was 9.98 (3.38). After adjusting for pre-test scores, the mean scores were 11.272 and 9.191, respectively (Table 2). The test of the within-group homogeneity regression coefficient showed no significant differences; the homogeneity regression coefficient hypothesis was valid (Table 3). An ANCOVA analysis revealed that the different health education materials had significantly different effects on the post-test score. After excluding the effect of the pre-test score, the result explained 4% of the variance of the types of teaching material with respect to the post-test score (partial eta square = 0.046) (Table 4). The EZ to read material improved oral health literacy scores of community older adults more than did the general text material. 

### 3.3. The MOHL-AQ Level Significantly Improved in EZ to Read Group

We divided the MOHL-AQ levels into three categorical groups based on the total score. The pre-test levels showed no significant difference between the groups (*p* = 0.525). Based on the comparison of the post-test levels between groups, the number of adequate scores among participants significantly increased in the EZ to read group. Both materials increased the MOHL-AQ levels within the group (*p* < 0.001). According to the data, health education improved all participants’ oral health literacy no matter what the material type was (significant pre-test and post-test difference within group), and the EZ to read material had a greater improving effect than the general text material. The post-test data significantly showed that more older adults reached an adequate level compared to the general text group (*p* value between groups in post-test = 0.014) (Table 5).

## 4. Discussion

Owing to the extension of life expectancy and decreasing fertility, the world is experiencing a continuous change in the age structure of the population. According to a report by the United Nations Department of Economic and Social Affairs, the number of adults aged 65 years and over reached 727 million people worldwide in 2020 and is projected to reach over 1.5 billion by 2025. The decline in fertility rates, coupled with global aging, has changed social and economic environments. Younger generations show higher educational levels and sustained rural-to-urban migration. In rural Taiwan, intergenerational co-residence has declined dramatically, and most older adults live alone, with their spouse, with their grandson [16], or with their non-relative caregiver. They lack a safety net of caretaking and material support from children and younger family members [17].

Taiwan is an aged society and will likely become a super-aged society in 2025, with the number of adults aged 65 and above crossing the 20% mark [18]. Longer life expectancy brings older adults a new chance to pursue long neglected passions and also contribute to their families and communities. The Taiwanese government set up community-based care centers that provide care services for older adults aged 65 and over nationwide. The services provided include health promotion activities, disability prevention activities, and lunch delivery, with the aim of maintaining their health, well-being, and quality of life.

Aging is accompanied by health problems, such as chronic metabolic disease, skeletomuscular degeneration, neurodegeneration, cognitive decline, multimorbidity, and oral health problems [19,20]. Oral health problems in older adults may affect the course and pathogenesis of a number of systemic diseases, such as cardiovascular disease, bacterial pneumonia, and diabetes mellitus [21]. Studies have provided evidence that lower health literacy in older adults leads to poor health outcomes [12]. Health literacy in rural populations is lower than that in urban populations. The difference between rural and urban areas can also be exacerbated by age, gender, education, and income [22]. It is important for health education materials to be readable to rural populations. Our study showed that more than 50% of older adults in the six rural areas were educated only up to the elementary school level. We did not include older adults who were not literate in this study (26% of initial sampling). Therefore, the specific educational material designed in the rural community must have symbols or pictures, in addition to text, so as to make them accessible to those with lower levels of literacy and weaker reading skills.

The concept of “EZ to read” was introduced to Taiwan from Europe. EZ to read functions as a tool to help people with reading difficulties. The use of EZ to read guidelines in hospital patient education materials showed a significant improvement in patients’ health literacy [10]. Improving health literacy in community populations requires special intervention tools [23]. This study utilizes EZ to read education material. The results indicated that health literacy was improved after health education regardless of the kind of material used. This reflected the importance of health education to older adult communities. In addition, the data provided revealed that the total number of older adults with an adequate oral health literacy level was significantly increased in the EZ to read group compared to the general text group. EZ to read material significantly improved not only the total score but the literacy level for older adults. It shed light on the importance of EZ to read methods applied to older adult education. We suggest that health professionals promote the EZ to read concept for use in educational materials in order to improve community health outcomes in rural areas.

This study had several potential limitations. The COVID-19 pandemic in Taiwan impeded activities held in community-based care centers. Our sample size was limited to six rural communities out of 46 (Wufeng: 22, Nantou Caotun: 24), and we could not enroll more participants because of the pandemic-induced lockdown. However, this exploratory study may offer some insight into the impact of EZ to read education material in rural areas. We suggest that the EZ to read concept be widely applied in older adult education in multiple fields. Furthermore, it is urgent to let community-based older adults become familiar with distance learning during pandemics. We believe that both distance education and e-education [24] are helpful to their staying socially healthy and continuing to learn.

## 5. Conclusions

Introducing the EZ to read model to the design of older adult health education material in rural areas significantly improved their oral health literacy. We suggest applying EZ to read concepts to widen the field of older adult education and to reduce illegibility-induced health knowledge disparities.

## Figures and Tables

**Table 1:** Demographic data of the participants in each group.

Variance		EZ to Read Material (*n*%)	General Text Material (*n*%)	*p* Value
Sex	Female	51 (70.8)	42 (73.7)	0.72
	Male	21 (29.2)	15 (26.3)	
Age (years)	60–64	4 (5.5)	3 (5.3)	0.361
	65–74	27 (37.5)	19 (33.4)	
	75–84	32 (44.5)	21 (36.8)	
	>85	9 (12.5)	14 (24.5)	
Educational level	Elementary	48 (66.7)	34 (59.7)	0.313
	Junior	11 (15.3)	11 (19.3)	
	Secondary	11 (15.3)	10 (17.5)	
	Vocational and technique school	2 (2.7)	0	
	Certificate and above	0	2 (3.5)	
Living status	Alone	12 (16.7)	7 (12.2)	0.522
	With a partner/spouse	9 (12.5)	3 (5.3)	
	With a partner/spouse and children	24 (33.3)	19 (33.3)	
	With children	24 (33.3)	25 (43.9)	
	With friend or relative	3 (4.2)	3 (5.3)	
Have a denture		58 (80.6)	46 (80.7)	0.983
Type of denture	Fixed	31 (54.4)	24 (51)	0.931
	Removable	17 (29.8)	14 (29.8)	
	Both	9 (15.8)	9 (19.2)	
Regular dental visit for denture adjustment	No	45 (62.5)	40 (70.2)	0.361
	Yes	27 (37.5)	17 (29.8)	

Chi-square test. EZ = easy.

**Table 2:** Descriptive statistics data of the MOHL-AQ post-test score between groups using different materials.

Group	*N*	Mean (SD)	^a^ Adjusted Mean (SE)
EZ to read material	72	11.222 (3.545)	11.272 (0.364)
General text material	57	9.982 (3.377)	9.919 (0.409)

^a^ Covariates appearing in the model are evaluated at the following values: average pre-test score = 5.287. SE = standard error. EZ = easy. SD = standard deviation.

**Table 3:** The test of homogeneity regression coefficient within groups.

Source	Type III Sum of Square	df	Mean Square	F
Group × pre-test score	32.67	1.00	32.67	3.48 n.s.
Error	1170.73	125.00	9.37	

Dependent variable: post-test score. df = degrees of freedom. n.s. = not significant.

**Table 4:** The ANCOVA analysis of the post-test score between different health education materials.

Source	Type III Sum of Squares	df	Mean Square	F	Sig.	Partial Eta Square
Pre-test score	328.024	1	328.024	34.345	<0.001	0.214
Group	58.196	1	58.196	6.093	0.015	0.046
Error	1203.403	126	9.551			
Corrected total	1580.326	128				

Dependent variable: post-test score. df = degrees of freedom.

**Table 5:** The difference between pre-test and post-test of MOHL-AQ levels within and between groups.

MOHL-AQ Level	EZ to Read Material (*n*%)		General Text Material (*n*%)		*p* Value between Group	
					Pre-Test	Post-Test
	Pre-test	Post-test	Pre-test	Post-test	0.525	0.014
Inadequate	66 (91.7)	23 (31.9)	50 (87.7)	23 (40.4)		
Marginal	4 (5.6)	8 (11.1)	3 (5.3)	15 (26.3)		
Adequate	2 (2.8)	41 (57)	4 (7)	19 (33.3)		
*p* value within group	*p* < 0.001		*p* < 0.001			

Chi-square test; MOHL-AQ: Mandarin version of the Oral Health Literacy Adult Questionnaire. Inadequate: score 0–9; marginal: score 10–11; adequate: score 12–17. EZ = easy.

## Data Availability

The data that support the findings of this study are available from the corresponding author, Yin-Hwa Shih, upon reasonable request.
